# (Con)Figuring out influence: a modified Delphi approach to configural diagramming to identify influential work system factors on emergency department disposition decision-making

**DOI:** 10.1080/00140139.2025.2606776

**Published:** 2026-01-05

**Authors:** Rachel A. Rutkowski, Pascale Carayon, Peter Hoonakker, Michael S. Pulia, Manish N. Shah, Brian W. Patterson, Nicole E. Werner

**Affiliations:** a Department of Industrial and Systems Engineering, University of Wisconsin (UW), Madison, WI, USA; b Berbee Walsh Department of Emergency Medicine, UW-Madison, Madison, WI, USA; c Department of Population Health Sciences, UW-Madison School of Medicine and Public Health, Madison, WI, USA; d Department of Anesthesiology, Vanderbilt University School of Medicine, Nashville, TN, USA; e Center for Research and Innovation in Systems Safety, Vanderbilt University Medical Center, Nashville, TN, USA

**Keywords:** Configuration, emergency department, disposition decision-making, work systems analysis, Delphi technique

## Abstract

The emergency department (ED) is characterised by varied demands. Yet, processes like ED disposition decision-making have been previously conceptualised as occurring similarly regardless of demand. The goal of this study was to develop a reproducible methodological approach to operationalising configuration by identifying the factors that most strongly shape the ED disposition decision-making process under conditions of low and high demand. Using a modified Delphi approach consisting of surveys sent to ED clinicians, we identified the influence of 32 work system elements on the disposition decision-making process under varied demands. Fifty-nine percent of elements were given similar ratings under low and high demand. Variations in ratings represent meaningful differences in the ED work system structure. Understanding how the influence of ED work system elements varies across the demand continuum can support the translation of descriptive findings into prescriptive insights that can inform and prioritise future research or system design.

**Practitioner Summary:**

This study leverages a modified Delphi approach to operationalise configuration to identify factors that strongly shape the emergency department disposition decision-making process under varied demands. Understanding the influence of work system elements across the demand continuum supports the translation of descriptive findings into prescriptive insights to prioritise future system design.

## 1. Introduction

Disposition decision-making determines the location to which a patient will transition upon the conclusion of their emergency department (ED) encounter (e.g. admission to the hospital, discharge home) (Agency for Healthcare Research and Quality 2011). Arriving at a disposition decision is typically a collaborative effort among ED clinicians, other healthcare professionals, patients, and care partners that involves gathering and processing patient data from multiple sources such as the electronic health record and the patient themselves (Calder et al. 2012; Capan et al. 2018). The decision-making process occurs across numerous sequential and concurrent tasks or sub-decision points (Calder et al. 2012; Probst et al. 2015; Rutkowski et al. 2022). These tasks may include soliciting information about the presenting complaint, conducting an evaluation of the patient, discussing clinical results with other clinicians, and diagnosing the patient (Calder et al. 2012; Capan et al. 2018).

The disposition decision is crucial in promoting patient safety and minimising healthcare costs, especially for high-volume patient populations at increased risk for suboptimal outcomes post-disposition such as older adults (Calder et al. 2010, 2012; Fernando et al. 2018; Ringer et al. 2018). Older adults have additional risks, needs, challenges, and considerations such as comorbidities that influence how they are cared for and dispositioned from the ED (Burton, Young, and Bernier 2014; Samaras et al. 2010). For example, for most patient populations, disposition planning primarily involves the consideration of proximal factors, such as a short-term recovery protocol. However, for an older adult be successful post-disposition, ED physicians often must leverage a hybrid approach that considers both proximal factors such as care partner support and long-term factors such as outpatient clinical support (Burton, Young, and Bernier 2014).

Fundamentally, disposition decision-making is a cognitive process that occurs as a result of ED clinicians navigating competing goals and variable demands (i.e. the resources required to execute the decision-making process) (Wears et al. 2010). Specifically, ED clinicians must manage and optimise for both acute goals with immediate with direct consequences such as efficiency and chronic goals such as patient safety and care quality. Reconciling acute and chronic goals often results in physicians striving to mitigate demands while navigating the tension between ‘providing optimal care to the individual patient and the need to provide care for multiple patients’.

The nuance of disposition decision-making is challenging to model. Existing frameworks do not explicitly consider how ED clinicians adapt their disposition decision-making behaviour under difference conditions (Calder et al. 2012; Capan et al. 2018; Rutkowski et al. 2020). Yet, drawing from the fields of naturalistic decision-making, distributed cognition, and work systems theory, we know that decision-makers compose, influence, and are influenced by the context within which they operate (Carayon 2009; Hendrick and Kleiner 2001; Hutchins 1995, 2000; Klein 2008; Lippa et al. 2017). Decision-making extends beyond ‘the skin or skull of an individual’ (Hutchins 2000, 1) and can be conceptualised as ‘an emergent property of people interacting with other actors and the environment’ (Lippa et al. 2017, 1036). Thus, to comprehensively model the disposition decision-making process, we must look beyond the individual decision-maker to characterise their broader context.

A sociotechnical work systems model, such as the Systems Engineering Initiative for Patient Safety (SEIPS) model, is well-equipped to identify the context within which the disposition decision-making process occurs (Carayon et al. 2006; Holden et al. 2013). The SEIPS model integrates concepts from healthcare quality and systems theory to describe the set of factors, referred to as elements, that define the system that influences a given process. Specifically, the SEIPS model identifies the *persons* who use *tools* to perform *tasks* within an *organisation*, *physical environment*, and broader *external environment*. These elements interact to produce processes and, ultimately, outcomes (Carayon et al. 2006, 2014).

Previous studies have identified numerous work system elements that influence the disposition decision-making process (Rutkowski et al. 2023). How these elements manifest within a work system seems to vary based on the presentation of other work system elements, which influences overall system demands (Holden et al. 2013; Rutkowski et al. 2025). Although it would be ideal to optimise each of these elements and elemental interactions through intervention or redesign, it is unrealistic to do so. Thus, a process that allows researchers to identify the elements that most strongly shape process performance, thereby having the greatest effect on outcomes, is needed to strategically target intervention and redesign efforts.

The concept of configuration asserts that ‘… only a subset of all possible [element] interactions is actually relevant in a given work process or situation … Thus, for a particular process or situation, one can distinguish a configuration of a finite number of relevant elements that interact to strongly shape the performance of that process’ (Holden et al. 2013, 6). Holden and colleagues go on to suggest that configuration can be used to ‘compare how two or more [hospital] units or organisations have configured their work system, by design or otherwise, for the same process’ (2013, 7). In response, previous work has used configuration to explore cross-boundary work systems (i.e. processes that extend across multiple work systems) (Werner et al. 2021), study the work system pre- and post-intervention implementation (Hay, Klonek, and Parker 2020), and understand the interactions, barriers and facilitators present within and among sub-systems involved in a process (Carman, Fray, and Waterson 2021). Therefore, based on Holden and colleagues’ definition of configuration, the suggested use cases, and previously published applications, it follows that configuration can be used to explore differences in performance of the *same* process within the *same* work system under different under varied demands.

Theoretically, the application of configuration and its utility in addressing complex work system questions is well-established (Carayon et al. 2006; Holden et al. 2013). Methodologically, though, there remains a dearth of information on how to operationalise the concept. Based on the definition, there are two key steps needed to perform a configural analysis: (1) identify the work system elements involved in shaping process performance and (2) determine the influence each work system element has on shaping process performance. Influence can be defined as the extent to which an element contributes to process performance and effects the observed process outcomes (Holden et al. 2013). Holden and colleagues note that configuration can be operationalised through the development of configural diagrams (i.e. visualisations of the most influential work system elements) using methods like ‘expert input, literature review, a voluntary reporting system, observations, interviews, surveys, and other[s]’ (2013, 7). These approaches have been widely applied and successful in addressing the first step in a configural analysis (Werner et al. 2021).

However, it is less clear how these methods can be used to determine the influence each work system element has on shaping process performance. Previous studies that have used the concept of configuration do not provide detailed descriptions of how they translated the attributes of configuration into tangible methods (Carman, Fray, and Waterson 2021; Hay, Klonek, and Parker 2020; Werner et al. 2021). Thus, the goal of this study was to use configuration to identify the factors that most strongly shape the ED disposition decision-making process for older adults under conditions of low and high demand and provide a reproducible methodological approach to creating configural diagrams. Specifically, our research questions include: What is the relative influence of ED work system elements on the disposition decision-making process for older adults? How does relative influence change under conditions of low and high demand? How can we operationalise the concept of configuration to capture and model the relative influence of elements on the disposition decision-making process?

## 2. Materials and methods

### 2.1. Study design

Configural analysis involves two key phases: identifying elements and identifying influence (Holden et al. 2013).

#### 2.1.1. Identifying elements

To identify the elements that compose the work system within which ED disposition decision-making occurs under conditions of high and low demands for older adults, we conducted a qualitative work system analysis. The intent of this study was to identify the work system elements that compose the ED work systems that influence and are influenced by the disposition decision-making process. This information provides the foundation needed to better understand the unique disposition considerations for older adults and how to (re)design ED work systems to make them more durable to demands while addressing the needs of the older adult population. The study consisted of a directed content analysis guided by the SEIPS model of contextual inquiry-based observations of older adults’ ED visits and semi-structured interviews with ED clinicians. From this study, we identified and characterised list of ED work system elements under conditions of high and low demands across all six work system components. Details of this motivating study can be found elsewhere (Rutkowski et al. 2025).

#### 2.1.2. Identifying influence

To gather data on the influence of these work system elements on the disposition decision-making process, we leveraged a modified Delphi approach. The Delphi technique is a ‘an iterative process used to collect and distil the judgments of experts using a series of questionnaires interspersed with feedback’ (Skulmoski, Hartman, and Krahn 2007, 2). It is a systematic approach to leveraging and making sense of experts’ opinions in the absence of a comprehensive theory or model (Helmer-Hirschberg 1967). The Delphi technique has a number of advantages including anonymity, iteration, controlled feedback, and statistical aggregation of group responses (Rowe and Wright 1999).

A traditional Delphi technique ‘follows a prescribed set of procedures that reflect both behavioural and statistical processes’. In its truest form, the Delphi technique could ‘be continuously iterated until consensus is determined to have been achieved’, meaning that there could be up to ‘n’ number of rounds of feedback (Hsu and Sandford 2007, 2). However, a review of studies that used the Delphi technique found that most studies achieved consensus in two or three rounds (Diamond et al. 2014). Further, response rates are known to decrease between rounds, especially among busy clinician participants (Graham 2010; Hasson, Keeney, and McKenna 2000; Keeney, Hasson, and McKenna 2006; McKenna 1994). As such, we determined that we would be able to glean the key benefits of a Delphi technique with a modified approach within two rounds without sacrificing the quality of the feedback and without overburdening participants.

There is no widely accepted definition or standard for establishing consensus in Delphi studies (Diamond et al. 2014; McKenna 1994). Previous work reports consensus thresholds ranging from 51% to 80%, with the majority coalescing around 75% (Keeney, Hasson, and McKenna 2006; McKenna 1994). Researchers caution against using an artificially high threshold for consensus as it may be inappropriate given the goals of the study and can lead to unnecessary participant burden (Loughlin and Moore 1979; McKenna 1994; McKenna and Hasson 2002). As such, we selected a consensus threshold of 65%. This threshold minimises participant burden while allowing us to draw meaningful conclusions about the perceptions of the majority of ED clinicians.

To gather data from clinicians, we designed and executed a two-round survey approach that asked participants to rate up to 32 elements on their influence on the disposition decision-making process for older adults under conditions of high and low demand. In consultation with emergency medicine subject matter experts, we distilled the original list of 40 work system elements characterised by Rutkowski et al. (2025) to 32 elements by combining sufficiently similar elements to reduce the survey completion time. Participants rated elements on a 5-point Likert scale from not influential to extremely influential (Appendix A) (Vagias 2006). Likert scales typically range from three to 13 points and there is no standard number of scale points. We selected a 5-point scale as it is familiar and perceived to be quick and easy to use (Preston and Colman 2000). A free textbox was included at the conclusion of each Likert block to provide participants the opportunity to indicate whether elements were missing or misrepresented.

Based on previous work (Rutkowski et al. 2025), in the introduction of the survey, we defined high demand as:

Consider a shift that is particularly chaotic and overwhelming. You’ve picked up multiple complex patients. The ED is under-staffed. You don’t have the time or resources to manage your patients in the way you’d like. You feel immense time pressure to get your patients out of the ED to make room for the many patients in the full waiting room.

We defined low demand as:

Consider a shift that is particularly manageable. You feel confident in your plans for each of your patients. You feel like you have all of the time and resources you need to manage your patients in the way you’d like. You feel only moderate time pressure to get patients out of the ED as the waiting room is not too full.

The survey was piloted with an emergency medicine physician and a qualitative researcher trained in cognitive psychology. This study took place at the ED of an academic medical centre with Level One trauma certification in the Midwest of the United States. The ED has 58 beds, three multiple purpose procedure rooms, a four-bed flexible care area, and two physician-in-triage/intake rooms. Approximately 70,000 adult and paediatric patients visit the ED each year, equating to over 300 patients per day. Older adult patients represent nearly 22% of ED visits annually. The patient population is considered high acuity with a 27.4% admission rate. The study was approved by the University Institutional Review Board, following a detailed review of study the protocol and materials.

### 2.2. Data collection

The surveys were built in and distributed through Qualtrics (Appendices B and C). ED clinicians were sent an initial mass recruitment email that contained a study information sheet and an invitation to participate, including the Qualtrics link to the first survey. The first survey was open for one month with a follow up reminder email sent to participants two weeks into the data collection period (Hsu and Sandford 2007). The second survey link was sent individually to the ED clinicians who participated in the first survey. Follow up reminder emails were sent every five days until all surveys were completed. Emails regarding the second survey included a summary of the participant’s responses and aggregate responses from all participants from the first survey, with the intent that participants would review this information ahead of or while taking the second survey. We collected data between May and June 2022. For their time, effort, and expertise, we offered participants a $100 honorarium in e-gift cards. Participants were paid $30 upon the completion of the first survey and $70 upon the completion of the second survey. The distribution of an honorarium was approved by the overseeing IRB.

There is no accepted method for determining the sample (i.e. panel) size or composition for a Delphi study. As such, we invited the entire ED clinician population to participate which included ED attendings, fellows, residents, and advanced practice providers [APP] at the medical centre where the study took place (n = 144) (Habibi, Sarafrazi, and Izadyar 2014; Keeney, Hasson, and McKenna 2006). Although numerous roles support the disposition decision-making process, such as nurses, we limited our participant population to the clinicians who are ultimately responsible for the final disposition decision (Calder et al. 2012; Rutkowski et al. 2020; Salwei et al. 2020).

### 2.3. Data analysis

We exported data from Qualtrics to Excel for further analysis and reviewed results as a research team. After each round of the survey, we aimed to determine whether at least 65% of ED clinician participants agreed on an element’s influence on the ED disposition decision-making process under conditions of high and low demand. We had initially intended to assess consensus at the individual Likert value level. For example, 65% or more of participants would have needed to report a single Likert value, on a scale from not influential to extremely influential, for an element to achieve consensus. However, after the first round of the survey, only four elements, two under high demand and two under low demand, achieved consensus.

As such, in consultation with subject matter experts in emergency medicine and cognitive psychology, after the second round of the survey, we elected to leverage a binned approach to consensus establishment where each bin represented a set of sufficiently similar Likert values (i.e. proportion within a range – unrestricted) (Diamond et al. 2014). This approach further relaxed the consensus threshold in so far as clinicians’ ratings generally needed to be within one Likert point of their peers’ ratings for consensus to be established. Specifically, the first bin consisted of the rating of ‘not influential’. The second bin consisted of the ratings ‘slightly influential’ and ‘somewhat influential’. The third bin consisted of the ratings ‘very influential’ and ‘extremely influential’.

Adjusting the specifications of the consensus retroactively led to a change in the number of elements that achieved consensus in the first round. Initially, establishing consensus using individual Likert values, four elements, two under high demand and two under low demand, achieved consensus. With the updated approach, three elements achieved consensus under conditions of low demand and 11 achieved consensus under conditions of high demand in the first round. Because we adjusted our approach after the completion of the second survey, participants were inadvertently surveyed on elements in the second round that had achieved consensus in the first round. Data from elements that achieved consensus in the first round with the updated consensus specifications were not considered in the analysis of data from the second survey, deferring to the results from the first survey.

For each work system element, we counted the number of responses within each bin and determined the percentage of responses that fell into each bin. If an element achieved consensus in the first round, it was not included for rating in the second-round survey. Elements that did not achieve consensus after the second-round survey were assigned the mode value identified from the second-round survey. Appendix D features an example of the enumeration we did for each element to determine whether consensus has been established.

To visualise our results, we generated two configural diagrams using a wire-framing software: one representing the ED work system that produces the disposition decision-making process under conditions of low demand and one under high demand. A configural diagram is a visual representation of the work system that features ‘the active and interacting work system’ elements for a process (Holden et al. 2013, 6). Each work system element is represented as a sphere and the influence that work system element has is represented by the size of the sphere. For simplicity, elements rated as ‘not influential’ were not included in the diagrams. Elements rated as ‘slightly influential’ or ‘somewhat influential’ were assigned a small sphere size. Elements rated as ‘very influential’ or ‘extremely influential’ were assigned a large sphere size. As is customary with work system visualisations, elements of the same component were grouped together (e.g. all person elements were displayed next to each other).

## 3. Results

### 3.1. Participants

The survey invitation was sent to 144 ED clinicians. We had a total of 33 participants (response rate: 33/144 = 23%). We achieved a 100% completion rate between the two surveys. Table 1 outlines participant demographics.

### 3.2. Survey results

Table 2 outlines the elements on which participants were surveyed and the assigned Likert value. Elements are grouped by work system component and elements rated similarly under conditions of low and high demand are highlighted in grey. Under conditions of low demand, three elements achieved consensus in the first survey and seven additional elements achieved consensus in the second survey. Under conditions of high demand, 11 elements achieved consensus in the first survey and an additional three elements achieved consensus the second survey.

One element was rated as ‘not influential’ under conditions of low and high demand. Twenty-six and 21 elements were rated as ‘somewhat influential’ or ‘slightly influential’ under conditions of low and high demand, respectively. Five and ten elements were rated as ‘very influential’ or ‘extremely influential’ under conditions of low and high demand, respectively. Nineteen of the 32 elements were given the same rating under both low and high demands. Of the 13 elements on which ratings differed, ratings always differed by one bin category. No free text comments were provided related to the content, quality, or quantity of the elements.

### 3.3. Configural diagram

**Figure 1.**
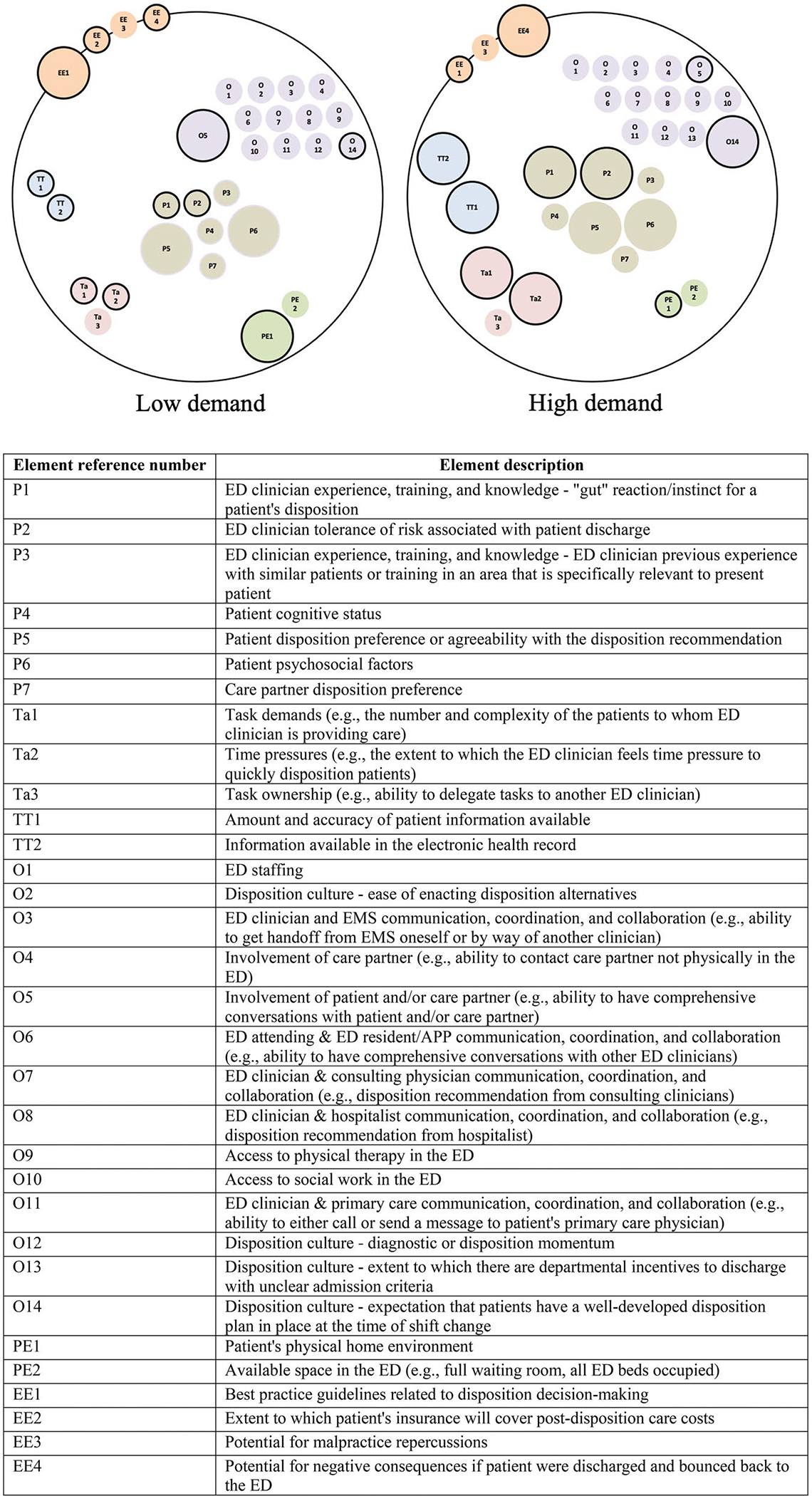
Configural diagrams representing the influential factors for the ED disposition decision-making process; EE = External environment; O = Organisation; P = Person; PE = Physical environment; Ta = Task; TT = Tools and technologies; The bold, black outline around bubbles identifies elements that varied in influence under conditions of low and high demand.

Figure 1 depicts the ED work system configurations that produce the disposition decision-making process under conditions of low and high demand. The sphere colours distinguish elements of the same component including person, tools and technology, task, organisation, physical environment, and external environment.

## 4. Discussion

The goal of the present study was to characterise the ED work system factors that most strongly shape the ED disposition decision-making process under conditions of low and high demand using the concept of configuration. Our findings highlight key differences in how ED clinicians perceive the influence of various work system elements on their performance of the disposition decision-making process in that 19 out of the 32 elements (59%) were given similar ratings under low and high demand. Variations in ratings between low and high demand represent meaningful differences in the ED work system structure that ultimately change how the disposition decision-making process is performed (Gorski et al. 2017). Understanding how the influence of ED work system elements varies across the demand continuum can translate descriptive findings into prescriptive insights that can inform and prioritise future research or system design (Holden et al. 2013).

Our study also introduced a novel application and approach to applying the concept of configuration through the creation of configural diagrams. The present work serves as a step-by-step guide on how to operationalise the concept of configuration, including key study design decision points and trade-offs.

### 4.1. Highly influential elements represent the greatest opportunity for improving process performance

We found that ED clinicians perceived most elements (59%) to have the same influence on ED disposition decision-making regardless of demand. This could indicate that the influence level of these elements may be immune to the dynamic, broader work system context. As such, intervening on elements perceived as consistently highly influential on the ED disposition decision-making process has the potential to have a meaningful and sustained effect on process performance under varied conditions.

Specifically, there were two elements that ED clinicians rated as highly influential under conditions of low and high demand: patient preference and psychosocial factors (e.g. access to transportation, living situation, ability to fulfil own future care needs). This suggests that these elements may represent areas in which system redesign is likely to be effective. With respect to patient preference for disposition location, previous studies have emphasised the pervasive time pressures and the frequent inability to fully engage patients and care partners in the ED (Adams et al. 2017; Daniels et al. 2018; Dyrstad, Testad, and Storm 2015; Lin et al. 2018; Pope et al. 2017; Rance et al. 2020; Schechtman et al. 2019; Stuck et al. 2017). Thus, developing and implementing efficient but robust mechanisms to elicit patient preference, such as the guidelines developed by Probst and colleagues, has the potential to greatly influence process performance (Probst et al. 2017). Likewise, research has previously identified a dearth of patient-related information available to clinicians in the ED (Nelson et al. 2013). As such, it follows that developing an information gathering tool or procedure to comprehensively capture pertinent psychosocial factors, making that information readily accessible, would meaningfully influence the ED disposition decision-making process under both low and high demands.

That said, although we have the ability to influence process performance through targeted intervention motivated by Delphi outputs, there is no guarantee that intervening on a highly influential element will yield exclusively favourable outcomes. Whether favourable outcomes occur due to intervention would depend on the quality of the intervention or redesign and how well it is implemented. The results presented in this study provide us with the anticipated magnitude of influence, not the anticipated outcome of intervention on an influential element.

### 4.2. Disparate ratings reflect meaningful differences in ED work system configuration

A subset of work system elements, at least one within each work system component, varied in their influence rating between low and high demand. This suggests that the ED work system configuration that produces the ED disposition decision-making process under conditions of low and high demand is fundamentally different.

Person elements, specifically those related to ED clinician risk tolerance and gestalt, were rated as more influential under conditions of high demand. Numerous studies have highlighted ED clinicians’ increased reliance on forms of rapid cognitive processing, especially when there is a heightened need to prioritise efficiency and an innate tendency to make strategic decisions to reduce demands (Calder et al. 2012; Daniels et al. 2018; Gorski et al. 2017; Nugus et al. 2011; Probst et al. 2015; Shanafelt et al. 2002; Turner et al. 2020; Wright et al. 2018).

Task elements, such as time pressure, were generally rated as more influential under conditions of high demand. The pervasive but variable nature of time pressure, the effect of patient census, and the number of high acuity patients on ED clinicians’ perception of overall demands is well-studied (Adams et al. 2017; Daniels et al. 2018; Dyrstad, Testad, and Storm 2015; Lin et al. 2018; Pope et al. 2017; Probst et al. 2015; Rance et al. 2020; Schechtman et al. 2019; Stuck et al. 2017). Time pressure is consistently reported as an inherent characteristic of the ED (Rutkowski et al. 2023; Wears et al. 2010). However, the motivation or source of time pressure seems to vary with demand. Under conditions of low demand, ED clinicians have reported attempting to maximise efficiency to reduce workload and ensure the ED is agile enough to respond to a sudden change in ED census (e.g. a mass-casualty event) (Rutkowski et al. 2023, 2025; Shanafelt et al. 2002; Turner et al. 2020). Under conditions of high demand, time pressure seems to be more focused on ethically managing ED census (Gorski et al. 2017). For instance, in their study exploring ED physician and general medicine physician perspectives on disposition decision-making, Daniels and colleagues note ‘an [emergency medicine] EM physician went on to explain competing demands in the ED: ‘If the patient is bordering for going home or coming in, those are the patients that I want to spend the least time on, because that is at the expense of patients who are really sick’ (742).

Tools and technology elements, including both the accuracy and accessibility of information, were rated as more influential under conditions of high demand. This could indicate that under high demands, clinicians rely more heavily on tools and technology to support their work. However, previous work has demonstrated that, due to issues of accuracy and accessibility, ED clinicians are often required to make disposition decisions without sufficient information (Nelson et al. 2013; Stiell et al. 2003). Thus, improving the accuracy and accessibility of information available in the ED has the potential to drastically shape the performance of the disposition decision-making process, specifically under high demands.

Of the organisation elements that varied in their ratings between high and low demand, the most notable is the involvement of patient and/or care partner. This element was rated as more influential under conditions of *low* demand. This could be due to the fact that, under low demand, ED clinicians have more time to engage in in-depth conversations, nuanced decisions, and additional pre-disposition coordination (Daniels et al. 2018; Rutkowski et al. 2023).

Likewise, the only physical environment element that varied was the patient’s home environment, with ED clinicians rating it as more influential under conditions of low demand. As described above, this could be related to the fact that under conditions of low demand, ED clinicians have an increased ability to have comprehensive conversations with patients and care partners.

The two external environment factors that varied with demand were best practice guidelines related to disposition decision-making and the potential for negative consequences should a patient incur a repeat ED following discharge. Previous work has noted the general lack of best practice guidelines, specifically for patients for whom a disposition decision is not obvious (Emerson et al. 2017; Lin et al. 2018; Probst et al. 2015). However, it is possible that best practice guidelines were rated higher under conditions of low demand because ED clinicians are likely to have more bandwidth to search for and assess the limited guidelines that may exist. The potential for negative consequences (e.g. medicolegal repercussions) should a patient promptly return to the ED is likely something that is always a consideration for ED clinicians (Siddique et al. 2018). However, under conditions of high demand it may be more of a consideration given that, although they provide the highest quality care possible given the circumstances, ED clinicians are more constrained in how they manage patients and make disposition decisions, which could lead to suboptimal outcomes.

### 4.3. Considerations for a modified Delphi approach to configuration

We demonstrated the feasibility of using a modified Delphi approach to produce configural diagrams that represent the same work system for the same process under two conditions. We made strategic study design decisions that led us to achieve favourable outcomes (e.g. 100% response rate on the second survey, short data collection period). However, there were numerous design choices that should be explored further.

#### 4.3.1. Study design and data collection considerations

Delphi approaches are notoriously ‘time-consuming and laborious’ as a result of the ‘iterative and sequential’ nature of the approach (Hasson, Keeney, and McKenna 2000; Hsu and Sandford 2007, 5; Keeney, Hasson, and McKenna 2006). Researchers often under-estimate the amount of time needed to administer the initial survey, obtain and analyse the data (e.g. follow up with non-respondents), and develop and distribute subsequent instruments to achieve consensus (Hsu and Sandford 2007; Keeney, Hasson, and McKenna 2006). Further, suboptimal response rates to web-based surveys among clinicians is a well-documented challenge in survey-based research (Cunningham et al. 2015). As such, to minimise the chance of encountering time-related challenges and encourage participation, we made a few key design decisions.

First, we established the number of rounds a priori. This strategy allowed us to scope our data collection period to a timeframe that would be reasonable for participants and the research team while still preserving the key characteristics of Delphi approaches (Hsu and Sandford 2007; Powell 2003). Researchers must consider the trade-offs between a higher proportion of survey elements that achieve consensus, achieved by increasing the number of survey rounds, and the time, cost and, participant fatigue that is likely to occur as the number of rounds increases (Graham 2010; Hasson, Keeney, and McKenna 2000; Keeney, Hasson, and McKenna 2006; McKenna 1994).

Second, given that there is no agreed-upon standard when selecting the consensus threshold, it is important to consider the purpose of the study. If the intent of the study is to make a high-impact or irreversible decision (e.g. update a department policy), a higher threshold may be warranted. If the intent of the study is to make a low-impact decision (e.g. determine tee-shirt colour) or assess perceptions, a lower threshold may be appropriate. Our intent with the present study is to assess what *most* ED clinicians think about the elements that most influence their disposition decision-making process. As such, we selected a consensus threshold of 65%. This threshold permitted some consensus latitude and reduced survey burden while allowing us to draw conclusions about the perceptions of the majority of ED clinicians.

Third, we sampled a population accustomed to participating in research. Our study occurred within the ED of an academic medical centre with an active commitment to research. As such, many of our participants likely had previous experience participating in research and were aware of the expectations, practices, and norms surrounding survey studies. This may have made them more likely to be compliant, leading all first-round participants to complete the second survey. Fourth, both surveys were short (~10 minutes), smartphone compatible, compensated, and contained topics that ED clinicians consider daily, all of which likely increased engagement (VanGeest, Johnson, and Welch 2007).

When interpreting the 23% overall response rate, there are a few factors to consider. Notably, the present qualitative study was designed to identify the *relative* influence elements have on the disposition decision-making process. To do so, we leveraged a modified Delphi technique through asynchronous surveys in place of more traditional consensus building techniques such as focus groups. Compared to focus groups, for example, our asynchronous survey data collection approach allowed us to gather more data from a wider range of clinician roles as we were not subject to the same time or resource constraints that accompany in-person data collection. Further, we collected data during a period where in-person access to clinicians was more restricted due to the COVID-19 pandemic. Further, our response rate is similar to that of related web-based, voluntary survey studies targeting clinicians (Cunningham et al. 2015). Given these considerations, our sample size is sufficient for achieving the goals of the present study.

With respect to data quality, there are several factors that should be considered. Pertaining to the feedback participants received alongside the second survey, we had no mechanism to ensure that participants reviewed and considered the feedback as they completed the second survey. We also had no mechanism to ensure complete anonymity. Because we aimed to recruit the entire population, participants were likely aware of other colleagues who were participating in the study. Although we have no reason to believe that participants collaborated, it is possible that participants discussed the surveys with one another (Keeney, Hasson, and McKenna 2006; McKenna 1994).

Related to the concept of consensus, it should be noted that ‘the extent to which participants agree with each other does not mean that consensus exists, nor does it mean that the ‘correct’ answer has been found’ (Keeney, Hasson, and McKenna 2006, 210). Participants may have all agreed that, in theory, element A is an extremely influential factor in shaping disposition decision-making. However, this may not align with how ED clinicians engage in the disposition decision-making process in their day-to-day work (Keeney, Hasson, and McKenna 2006). In other words, there may be a misalignment between ED clinicians’ work as imagined (i.e. what ED clinicians ideally do), work as perceived (i.e. what ED clinicians think they do), and work as done (i.e. what ED clinicians actually do) (Leplat 1989). Future work may consider using more just-in-time, embedded, or observational cognitive approaches to assess the elements that acutely shape ED clinicians’ disposition decision-making processes.

Inherently, a Delphi approach conceals dissenting or disparate opinions (Keeney, Hasson, and McKenna 2006). As part of the consensus building process, participants may have felt inclined to change their responses ‘because of a possible mistaken belief that the views expressed by the majority of the panel must be right’ or ‘they see that someone else has identified a more relevant issue that they had not thought of’ (Keeney, Hasson, and McKenna 2006, 210). As such, although we achieved consensus, our findings may not fully reflect the varied opinions or perspectives of all ED clinicians. Tools like member checking or approaches that honour the variability of individual experiences such as case studies or focus groups could be used to assess the extent to which our findings reflect the full range of ED clinician experiences.

Finally, practically, the default selection in the Qualtrics software was ‘1 - Not influential’. Defaults are known to influence participants’ opinions. Participants did still need to click into each question for the question to be marked ‘complete’. However, the extreme nature of the default selection likely biased participants less than a neutral default (Chimi 2022).

#### 4.3.2. Data analysis considerations

We had initially intended to establish consensus using individual Likert values. However, upon reviewing the results, we determined that consensus at the Likert value level introduced unnecessary consensus burden given that the goal of the study was to assess relative influence (Loughlin and Moore 1979; McKenna 1994; McKenna and Hasson 2002). With the ultimate goal of developing configural diagrams, we found no meaningful benefit in differentiating between ‘4– very influential’ and ‘5 – extremely influential’, for example, as both indicate a strong influence on disposition decision-making. As such, we transitioned to a bin approach where each bin represented a set of Likert values (Diamond et al. 2014). The first bin consisted of the rating of ‘not influential’. The second bin consisted of the ratings ‘slightly influential’ and ‘somewhat influential’. The third bin consisted of the ratings ‘very influential’ and ‘extremely influential’.

We defined the bins in this manner based on the natural delineation points along the influence spectrum. This approach minimised survey burden by relaxing the consensus threshold and reduced clutter on the configural diagrams by limiting the number of sphere sizes. However, there are other approaches that could be used to analyse the data through different binning structures which could change how consensus is established and how elements are depicted in the configural diagrams. Researchers should consider which approach supports their objectives.

With respect to the number of elements that achieved consensus, only a subset of elements achieved consensus after the second survey. Rather than engaging in subsequent survey rounds with the goal of achieving consensus on additional elements, elements that did not achieve consensus after the second-round survey were assigned the mode value identified from the second-round survey. Although this approach reduced the number of elements on which we *could* have established consensus, we prioritised survey participant experience by limiting the survey rounds and were still able to generate the data needed to support the development of configural diagrams (Loughlin and Moore 1979; McKenna 1994; McKenna and Hasson 2002).

That said, it is important to consider the unique benefit of the modified Delphi approach compared to other approaches to consensus establishment and data collection. Compared to other approaches to consensus establishment such as focus groups, we generated more concrete data regarding consensus. While focus groups tend to generate a large quantity of data, it can be challenging to assess the extent to which participants agree on a given topic without more structured rating. Further, focus groups can be time consuming to conduct and challenging to coordinate with clinician participants. Through the two survey rounds, we were able to quantify the extent to which consensus was achieved and participants were able to complete the surveys at their convenience.

A two-stage rating process generated additional data about the broader nature and transferability of an element’s influence. Elements that achieved consensus likely represent factors that are more universal in their influence. These elements represent areas where work system intervention is likely to be successful and most impactful given that most clinicians agree on their importance (Holden et al. 2013). Elements on which we did not achieve consensus may indicate divergence in ED clinicians’ practices such that certain elements influence their decision-making more than others (Westert and Groenewegen 1999). These individual differences may indicate where ED work system design may need to be more adaptive and warrant further investigation to fully explicate why these disparate perspectives exist.

Based on work systems theory, we assumed that all work system elements within the configural diagram interact (Carayon et al. 2006, 2014; Holden et al. 2013). However, work systems theory suggests that some interactions may be more influential than others on shaping process performance (Holden et al. 2013; Holden and Carayon 2021). We explored the work system at the elemental level, rather than the interaction level, outlining which elements influence the disposition decision-making process and to what extent. However, the interconnectedness of work system elements and how those interactions shape process performance is important to consider. Future work should aim to recruit a mechanism such as epistemic network analysis or co-occurrence analysis to capture and depict why and how elements interact to provide the most robust description of the work system configurations.

Likewise, with respect to the visualisations, configural diagrams generally include lines that connect interacting work system elements. However, previously published papers using configural diagramming, including the paper from which the method originates, do not provide a description of how to strategically position lines to connect interacting elements given that all elements within a work system interact with one another to some extent (Hay, Klonek, and Parker 2020; Holden, Schubert, and Mickelson 2015; Holden et al. 2013; Holden and Carayon 2021; Werner et al. 2021). In their 2021 paper, Holden and Carayon point out that configural ‘diagrams are not meant to be fully exclusive; they are better suited to show only the most relevant or consequential factors or interactions’ (7). Yet, how to determine the most relevant or consequential interactions is still unclear. Thus, to avoid overcomplicating and cluttering our configural diagrams, we did not include lines connecting interacting elements.

### 4.4. Limitations

The results from this study should be interpreted considering several limitations. First, data were collected at one academic teaching hospital and focused on older adults. Aspects of the results may be transferable to other patient populations with unique disposition considerations, such as children with medical complexity. However, the results may not be representative of disposition decision-making at other types of institutions or for every patient population. Second, in developing and executing the modified Delphi approach, we made numerous design choices such as the size of the participant panel, the consensus threshold, the number of survey rounds, the nature of the survey, and the binning of the results. For many of these study specifications, there is no universally agreed-upon standard. Yet, each has the potential to greatly influence participant experience and study results. The present study represents one combination of specifications that were selected to address our goals. However, future work is needed to ascertain the influence each specification may have on study outcomes and how to optimise specifications for configural diagramming.

## 5. Conclusion

The present study extends our understanding of how the ED work system shapes the performance of the disposition decision-making process and also introduces a novel approach to operationalising configuration. Practically, our findings have the potential to inform the adaptive and reflexive design of interventions and system structures to support the disposition decision-making. Methodologically, our work expands the work systems analysis toolkit by providing a methodological playbook for configural diagramming, which provides researchers with a mechanism to identify the most influential work systems elements in shaping process performance. The ability to identify these influential elements has the potential to increase the effectiveness and efficiency of work systems research, thereby permitting the translation of rigorous work system analyses into actionable change that will affect patient, care partner, clinician, and organisational outcomes.

## Figures and Tables

**Table 1.** Participant demographics.

Demographic variable	Value

**Age (years)**	
Average (range)	33 (27–47)
**Gender (count)**	
Female	16 (49%)
**Role (count)**	
ED attending	12 (37%)
ED fellow	1 (3%)
Intern physician (PgY-1)	5 (15%)
2nd year resident physician (PgY-2)	7 (21%)
3rd year resident physician (PgY-3)	1 (3%)
ED APP	7 (21%)
**Tenure (years)**	
Average (range)	4 (1–25)
**Estimated percentage of patient load aged >65 years old (%)**	
Average (range)	54 (30–75)

**Table 2.** Summary of element ratings.

component	Element	Low demand influence rating	High demand influence rating
P	ED clinician experience, training, and knowledge – ‘gut’ reaction/ instinct for a patient’s disposition	Somewhat or slightly influential**	Very or extremely influential
P	ED clinician tolerance of risk associated with patient discharge	Somewhat or slightly influential*	Very or extremely influential
P	ED clinician experience, training, and knowledge – ED clinician previous experience with similar patients or training in an area that is specifically relevant to present patient	Somewhat or slightly influential	Somewhat or slightly influential
P	Patient cognitive status	Somewhat or slightly influential	Somewhat or slightly influential
P	Patient disposition preference or agreeability with the disposition recommendation	Very or extremely influential	Very or extremely influential
P	Patient psychosocial factors	Very or extremely influential**	Very or extremely influential*
P	Care partner disposition preference	Somewhat or slightly influential	Somewhat or slightly influential*
Ta	Task demands (e.g. the number and complexity of the patients to whom ED clinician is providing care)	Somewhat or slightly influential	Very or extremely influential
Ta	Time pressures (e.g. the extent to which the ED clinician feels time pressure to quickly disposition patients)	Somewhat or slightly influential	Very or extremely influential
Ta	Task ownership (e.g. ability to delegate tasks to another ED clinician)	Somewhat or slightly influential*	Somewhat or slightly influential*
TT	Amount and accuracy of patient information available	Somewhat or slightly influential	Very or extremely influential
TT	Information available in the electronic health record	Somewhat or slightly influential	Very or extremely influential
o	ED staffing	Somewhat or slightly influential	Somewhat or slightly influential*
o	Disposition culture – ease of enacting disposition alternatives	Somewhat or slightly influential*	Somewhat or slightly influential*
o	ED clinician and Ems communication, coordination, and collaboration (e.g. ability to get handoff from Ems oneself or by way of another clinician)	Somewhat or slightly influential	Somewhat or slightly influential*
o	Involvement of care partner (e.g. ability to contact care partner not physically in the ED)	Somewhat or slightly influential**	Somewhat or slightly influential*
o	Involvement of patient and/or care partner (e.g. ability to have comprehensive conversations with patient and/or care partner)	Very or extremely influential	Somewhat or slightly influential
o	ED attending & ED resident/APP communication, coordination, and collaboration (e.g. ability to have comprehensive conversations with other ED clinicians)	Somewhat or slightly influential	Somewhat or slightly influential*
o	ED clinician & consulting physician communication, coordination, and collaboration (e.g. disposition recommendation from consulting clinicians)	Somewhat or slightly influential**	Somewhat or slightly influential*
o	ED clinician & hospitalist communication, coordination, and collaboration (e.g. disposition recommendation from hospitalist)	Somewhat or slightly influential	Somewhat or slightly influential**
o	Access to physical therapy in the ED	Somewhat or slightly influential	Somewhat or slightly influential
o	Access to social work in the ED	Somewhat or slightly influential	Somewhat or slightly influential
o	ED clinician & primary care communication, coordination, and collaboration (e.g. ability to either call or send a message to patient’s primary care physician)	Somewhat or slightly influential**	Somewhat or slightly influential**
o	Disposition culture – diagnostic or disposition momentum	Somewhat or slightly influential**	Somewhat or slightly influential
o	Disposition culture – extent to which there are departmental incentives to discharge patients with unclear admission criteria	Not influential	Somewhat or slightly influential**
o	Disposition culture – expectation that patients have a well-developed disposition plan in place at the time of shift change	Somewhat or slightly influential	Very or extremely influential
PE	Patient’s physical home environment	Very or extremely influential	Somewhat or slightly influential
PE	Available space in the ED (e.g. full waiting room, all ED beds occupied)	Somewhat or slightly influential**	Somewhat or slightly influential*
EE	Best practice guidelines related to disposition decision-making	Very or extremely influential	Somewhat or slightly influential*
EE	Extent to which patient’s insurance will cover post-disposition care costs	Somewhat or slightly influential	Not influential
EE	Potential for malpractice repercussions	Somewhat or slightly influential	Somewhat or slightly influential
EE	Potential for negative consequences if patient were discharged and bounced back to the ED	Somewhat or slightly influential	Very or extremely influential

EE = External environment; o = organisation; P = Person; PE = Physical environment; Ta =Task; TT =Tools and technology; greyed boxes = elements provided the same rating under low and high demand; White boxes = elements rated differently under low and high demand; not influential = Likert value equal to one; somewhat or slightly influential = Likert value equal to two or three; Very or extremely influential = Likert value equal to four or five.

* = Elements that achieved consensus in the first round of the survey;

** = Elements that achieved consensus in the second round of the survey.

## Appendix A: Likert scale of level of influence

1. Not influential
2. Slight influential
3. Somewhat influential
4. Very influential
5. Extremely influential

## Appendix A reference

Vagias, Wade M. 2006. *Likert-Type Scale Response Anchors*. Clemson International Institute for Tourism & Research Development, Department of Parks, Recreation and Tourism Management. Clemson University.

## Appendix B: Delphi survey 1

### Delphi survey 1

Start of Block: Default Question Block

We would like you to complete this survey evaluating the factors that influence your disposition decision-making for older adults (older than 65 years) during two types of shifts: (1) chaotic and overwhelming and (2) manageable.

In the survey we will ask for your job title, your gender, and your age. This data is confidential and will only be used to conduct analyses at the group level (e.g. resident physicians vs. attending physicians).

The survey will take approximately 10 minutes to fill out. This is the first of two surveys you will receive. The second survey will be sent to your email in approximately two weeks.

Thank you for your participation!

What is your job title?

- Intern physician (PGY-1) (1)
- 2nd year resident physician (PGY-2) (2)
- 3rd year resident physician (PGY-3) (3)
- Fellow (4)
- Attending physician (5)
- Advanced practice provider (e.g. PA) (6)
- Other – please specify (7) ______________________________________________

How many total years have you worked in this role, both at UW and other institutions?

_____________________________________________________________________________________________________________________

What is your gender?

- Male (1)
- Female (2)
- Non-binary/third gender (3)
- Prefer not to say (4)

What is your age?

____________________________________________________________

Please estimate the percentage of your patient load that is aged 65 years old or older

**Table:**

	0	10	20	30	40	50	60	70	80	90	100

% of your patient load that is aged 65+											
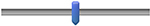

Consider a shift that is particularly chaotic and overwhelming. You are managing multiple complex and high acuity patients. The emergency department (ED) is under-staffed. You don’t have the time or resources to manage your patients in the way you’d like. You feel immense time pressure to get your patients out of the ED as the waiting room is full and wait times are long.

Please provide a rating (1 = not influential; 5 = extremely influential) for each factor on the extent to which it influences your disposition decision-making process for older adults in the ED during a **chaotic and overwhelming** shift.

**Table:**

	Not influential 1	Slightly influential 2	Somewhat influential 3	Very influential 4	Extremely influential 5

Best practice guidelines related to disposition decision-making ()					
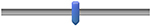
Extent to which patient’s insurance will cover subsequent care costs ()					
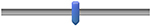
Potential for malpractice repercussions ()					
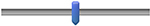
Potential for negative consequences if your patient were discharged and bounced back to the ED ()					
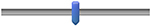
Patient’s physical home environment (e.g. stairs to get into house, bathroom only on second floor) ()					
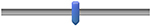
ED crowding (e.g. use of hallway beds, boarding, number of patients in waiting room) ()					
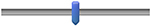
ED staffing (e.g. number of nurses) ()					
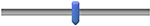
Amount and accuracy of patient information available to you (e.g. baseline status, living situation) ()					
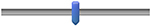
Information available in the electronic health record (e.g. medical history, medications) ()					
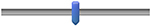
The number and complexity of the patients to whom you’re providing care ()					
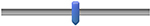
The extent to which you feel time pressure to quickly disposition patients (e.g. to see waiting room patients, manage ED patient flow, minimise length of stay) ()					
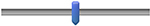
Ease of enacting disposition alternatives (e.g. amount of follow-up care coordination required to discharge, number of phone calls required to admit) ()					
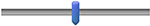
Ability to delegate tasks to another ED clinician (e.g. ED nurse calling patient’s family and reporting back) ()					
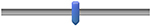
Ability to get handoff from Ems yourself or by way of another ED clinician ()					
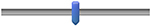
Ability to contact care partner not physically in the ED (e.g. by phone) ()					
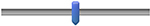
Ability to have comprehensive conversations with patient and/or care partner ()					
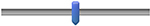
Ability to have comprehensive conversations with other ED clinicians ()					
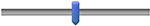
Recommendation from consulting clinicians (e.g. ortho, neuro) ()					
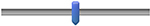
Recommendation from hospitalist ()					
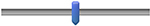
Access to physical therapy in the ED ()					
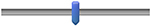
Access to social work in the ED ()					
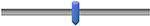
Ability to either call or send an inbasket message to patient’s primary care physician ()					
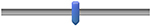
Diagnostic or disposition momentum (e.g. inclination to do what has been done before for the patient) ()					
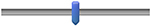
Extent to which there are departmental incentives to discharge “grey-zone” patients ()					
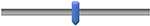
Expectation that patients have a well-developed disposition plan in place at the time of shift change ()					
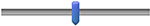
Your “gut” reaction/instinct for a patient’s disposition ()					
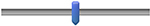
Your personal comfort discharging a patient with a high risk of adverse outcomes ()					
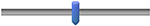
Your previous experience with similar patients or training in an area that is specifically relevant to your present patient ()					
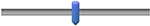
Patient cognitive status ()					
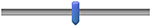
Patient disposition preference or agreeability with your disposition recommendation ()					
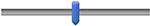
Patient psychosocial factors (e.g. access to transportation, living situation, ability to fulfil own future care needs) ()					
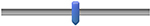
Care partner disposition preference ()					
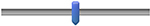

Do you have any comments about disposition decision-making for older adult patients during a **chaotic and overwhelming shift**?

Consider a shift that is manageable. You are taking care of a comfortable number of patients and very few are high acuity. You feel confident in your plans for each of your patients. You feel like you have all of the time and resources you need to manage your patients in the way you’d like. You feel very little time pressure to get patients out of the ED as the waiting room is nearly empty and wait times are short.

Please provide a rating (1 = not influential; 5 = extremely influential) for each factor on the extent to which it influences your disposition decision-making process for older adults in the ED during a **manageable** shift.

**Table:**

	Not influential 1	Slightly influential 2	Somewhat influential 3	Very influential 4	Extremely influential 5

Best practice guidelines related to disposition decision-making ()					
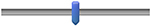
Extent to which patient’s insurance will cover subsequent care costs ()					
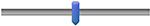
Potential for malpractice repercussions ()					
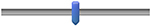
Potential for negative consequences if your patient were discharged and bounced back to the ED ()					
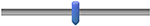
Patient’s physical home environment (e.g. stairs to get into house, bathroom only on second floor) ()					
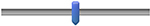
ED crowding (e.g. use of hallway beds, boarding, number of patients in waiting room) ()					
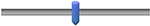
ED staffing (e.g. number of nurses) ()					
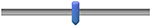
Amount and accuracy of patient information available to you (e.g. baseline status, living situation) ()					
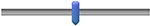
Information available in the electronic health record (e.g. medical history, medications) ()					
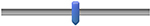
The number and complexity of the patients to whom you’re providing care ()					
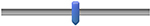
The extent to which you feel time pressure to quickly disposition patients (e.g. to see waiting room patients, manage ED patient flow, minimise length of stay) ()					
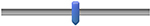
Ease of enacting disposition alternatives (e.g. amount of follow-up care coordination required to discharge, number of phone calls required to admit) ()					
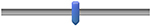
Ability to delegate tasks to another ED clinician (e.g. ED nurse calling patient’s family and reporting back) ()					
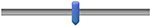
Ability to get handoff from Ems yourself or by way of another ED clinician ()					
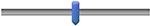
Ability to contact care partner not physically in the ED (e.g. by phone) ()					
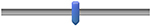
Ability to have comprehensive conversations with patient and/or care partner ()					
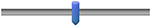
Ability to have comprehensive conversations with other ED clinicians ()					
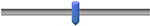
Recommendation from consulting clinicians (e.g. ortho, neuro) ()					
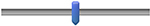
Recommendation from hospitalist ()					
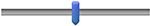
Access to physical therapy in the ED ()					
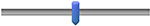
Access to social work in the ED ()					
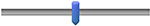
Ability to either call or send an inbasket message to patient’s primary care physician ()					
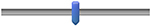
Diagnostic or disposition momentum (e.g. inclination to do what has been done before for the patient) ()					
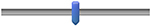
Extent to which there are departmental incentives to discharge “grey-zone” patients ()					
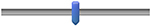
Expectation that patients have a well-developed disposition plan in place at the time of shift change ()					
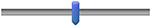
Your “gut” reaction/instinct for a patient’s disposition ()					
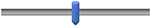
Your personal comfort discharging a patient with a high risk of adverse outcomes ()					
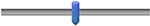
Your previous experience with similar patients or training in an area that is specifically relevant to your present patient ()					
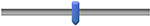
Patient cognitive status ()					
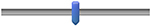
Patient disposition preference or agreeability with your disposition recommendation ()					
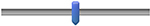
Patient psychosocial factors (e.g. access to transportation, living situation, ability to fulfil own future care needs) ()					
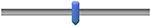
Care partner disposition preference ()					
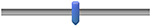

Do you have any comments about disposition decision-making for older adult patients during a **manageable** shift?

End of Block: Default Question Block

Start of Block: Follow Up Info

Please enter the first letter of your last name the last four digits of your phone number. For example, if someone’s telephone number is 608–678-1234 and last name is Johnson, they would enter 1234 J. This will serve as your unique, anonymous identifier to link your responses from the first and second survey.

Please enter your UW-Health email address. We will send the second survey link to this email.

Upon completion of this survey, you will be sent a $30 Amazon e-gift card. Please provide the email address to which you would like your e-gift card sent.

End of Block: Follow Up Info

## Appendix C: Delphi survey 2

### Delphi survey 2

Start of Block: Default Question Block

Thank you so much for your participation in the first Patient Safety Learning Lab survey exploring the factors that most influence your disposition decision-making process for older adults in the ED. We had a total of 33 participants complete the first survey.

In analysing the results from the first survey, we found that you and your fellow ED clinicians agreed on the influence of the following factors on the disposition decision-making process during a **chaotic and overwhelming shift**:

- ED crowding
- Patient psychosocial factors

We found that you and your fellow ED clinicians agreed on the influence of the following factors on the disposition decision-making process during a **manageable shift**:

- Patient’s home environment
- The extent to which you feel time pressure to quickly disposition patients

There were a total of 30 factors for a chaotic and overwhelming shift and 30 factors for a manageable shift on which you and your fellow ED clinicians had disparate perspectives on their influence on the disposition decision-making process.

In this second survey, we would ask that you:

1. Briefly review your responses as well as the aggregate responses from the first survey (sent to you via email)
2. Re-rate the factors on which you and your fellow ED clinicians had dissimilar ratings. You may choose to change your rating from the first round or keep it the same.

The survey will take approximately 10 minutes to fill out.

Thank you for your participation!

Consider a shift that is particularly chaotic and overwhelming. You are managing multiple complex and high acuity patients. The emergency department (ED) is under-staffed. You don’t have the time or resources to manage your patients in the way you’d like. You feel immense time pressure to get your patients out of the ED as the waiting room is full and wait times are long.

Please provide a rating (1 = not influential; 5 = extremely influential) for each factor on the extent to which it influences your disposition decision-making process for older adults in the ED during a **chaotic and overwhelming** shift.

**Table:**

	Not influential 1	Slightly influential 2	Somewhat influential 3	Very influential 4	Extremely influential 5

Best practice guidelines related to disposition decision-making ()					
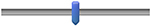
Extent to which patient’s insurance will cover subsequent care costs ()					
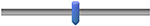
Potential for malpractice repercussions ()					
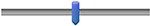
Potential for negative consequences if your patient were discharged and bounced back to the ED ()					
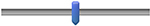
Patient’s physical home environment (e.g. stairs to get into house, bathroom only on second floor) ()					
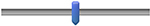
ED staffing (e.g. number of nurses) ()					
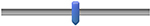
Amount and accuracy of patient information available to you (e.g. baseline status, living situation) ()					
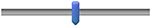
Information available in the electronic health record (e.g. medical history, medications) ()					
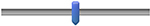
The number and complexity of the patients to whom you’re providing care ()					
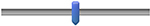
The extent to which you feel time pressure to quickly disposition patients (e.g. to see waiting room patients, manage ED patient flow, minimise length of stay) ()					
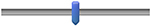
Ease of enacting disposition alternatives (e.g. amount of follow-up care coordination required to discharge, number of phone calls required to admit) ()					
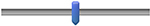
Ability to delegate tasks to another ED clinician (e.g. ED nurse calling patient’s family and reporting back) ()					
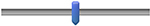
Ability to get handoff from Ems yourself or by way of another ED clinician ()					
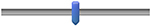
Ability to contact care partner not physically in the ED (e.g. by phone) ()					
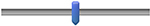
Ability to have comprehensive conversations with patient and/or care partner ()					
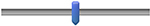
Ability to have comprehensive conversations with other ED clinicians ()					
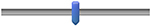
Recommendation from consulting clinicians (e.g. ortho, neuro) ()					
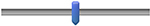
Recommendation from hospitalist ()					
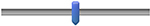
Access to physical therapy in the ED ()					
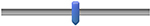
Access to social work in the ED ()					
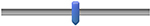
Ability to either call or send an inbasket message to patient’s primary care physician ()					
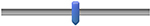
Diagnostic or disposition momentum (e.g. inclination to do what has been done before for the patient) ()					
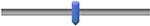
Extent to which there are departmental incentives to discharge “grey-zone” patients ()					
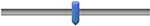
Expectation that patients have a well-developed disposition plan in place at the time of shift change ()					
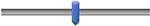
Your “gut” reaction/instinct for a patient’s disposition ()					
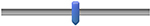
Your personal comfort discharging a patient with a high risk of adverse outcomes ()					
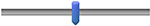
Your previous experience with similar patients or training in an area that is specifically relevant to your present patient ()					
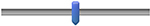
Patient cognitive status ()					
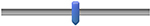
Patient disposition preference or agreeability with your disposition recommendation ()					
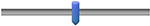
Care partner disposition preference ()					
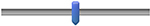

Do you have any comments about disposition decision-making for older adult patients during a **chaotic and overwhelming shift**?

Consider a shift that is manageable. You are taking care of a comfortable number of patients and very few are high acuity. You feel confident in your plans for each of your patients. You feel like you have all of the time and resources you need to manage your patients in the way you’d like. You feel very little time pressure to get patients out of the ED as the waiting room is nearly empty and wait times are short.

Please provide a rating (1 = not influential; 5 = extremely influential) for each factor on the extent to which it influences your disposition decision-making process for older adults in the ED during a **manageable** shift.

**Table:**

	Not influential 1	Slightly influential 2	Somewhat influential 3	Very influential 4	Extremely influential 5

Best practice guidelines related to disposition decision-making ()					
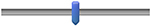
Extent to which patient’s insurance will cover subsequent care costs ()					
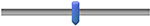
Potential for malpractice repercussions ()					
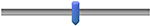
Potential for negative consequences if your patient were discharged and bounced back to the ED ()					
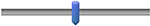
ED crowding (e.g. use of hallway beds, boarding, number of patients in waiting room) ()					
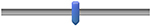
ED staffing (e.g. number of nurses) ()					
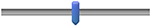
Amount and accuracy of patient information available to you (e.g. baseline status, living situation) ()					
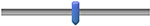
Information available in the electronic health record (e.g. medical history, medications) ()					
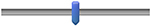
The number and complexity of the patients to whom you’re providing care ()					
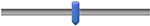
Ease of enacting disposition alternatives (e.g. amount of follow-up care coordination required to discharge, number of phone calls required to admit) ()					
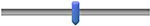
Ability to delegate tasks to another ED clinician (e.g. ED nurse calling patient’s family and reporting back) ()					
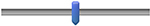
Ability to get handoff from Ems yourself or by way of another ED clinician ()					
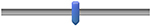
Ability to contact care partner not physically in the ED (e.g. by phone) ()					
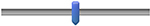
Ability to have comprehensive conversations with patient and/or care partner ()					
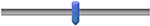
Ability to have comprehensive conversations with other ED clinicians ()					
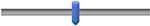
Recommendation from consulting clinicians (e.g. ortho, neuro) ()					
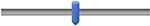
Recommendation from hospitalist ()					
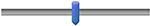
Access to physical therapy in the ED ()					
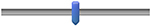
Access to social work in the ED ()					
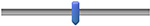
Ability to either call or send an inbasket message to patient’s primary care physician ()					
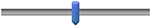
Diagnostic or disposition momentum (e.g. inclination to do what has been done before for the patient) ()					
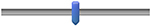
Extent to which there are departmental incentives to discharge “grey-zone” patients ()					
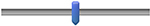
Expectation that patients have a well-developed disposition plan in place at the time of shift change ()					
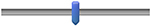
Your “gut” reaction/instinct for a patient’s disposition ()					
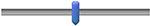
Your personal comfort discharging a patient with a high risk of adverse outcomes ()					
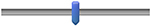
Your previous experience with similar patients or training in an area that is specifically relevant to your present patient ()					
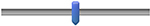
Patient cognitive status ()					
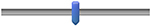
Patient disposition preference or agreeability with your disposition recommendation ()					
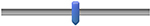
Patient psychosocial factors (e.g. access to transportation, living situation, ability to fulfil own future care needs) ()					
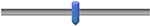
Care partner disposition preference ()					
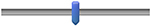

Do you have any comments about disposition decision-making for older adult patients during a **manageable** shift?

End of Block: Default Question Block

Start of Block: Follow Up Info

Please enter the first letter of your last name the last four digits of your phone number. For example, if someone’s telephone number is 608–678-1234 and last name is Johnson, they would enter 1234 J. This will serve as your unique, anonymous identifier to link your responses from the first and second survey.

Upon completion of this survey, you will be sent a $70 Amazon e-gift card. Please provide the email address to which you would like your e-gift card sent.

End of Block: Follow Up Info

## Appendix D: Example Delphi calculations

Below is an example of the calculations done for each element surveyed. These values relate to the element ‘best practice guidelines related to disposition decision-making’.

**Table:**

Summary statistic	Element summary

# of 1s	1
# of 2s	10
# of 3s	12
# of 4s	4
# of 5s	6
# of non-responses	0
% of 1s	3.03
% of 2s	30.30
% of 3s	36.36
% of 4s	12.12
% of 5s	18.18
Relative % of 1s	3.03
Relative % of 2s and 3s	66.67
Relative % of 4s and 5s	30.3
Mode	3

## References

[R1] Adams E, Goyder C, Heneghan C, Brand L, and Ajjawi R. 2017. “Clinical Reasoning of Junior Doctors in Emergency Medicine: A Grounded Theory Study.” Emergency Medicine Journal: EMJ 34 (2): 70–75. doi:10.1136/emermed-2015-205650. 27340131

[R2] Agency for Healthcare Research and Quality. 2011. “Disposition of Patient.” Accessed 06 November 22. https://ushik.ahrq.gov/ViewItemDetails?system=mdr&itemKey=65837000

[R3] Burton JH, Young J, and Bernier CA. 2014. “The Geriatric ED: Structure, Patient Care, and Considerations for the Emergency Department Geriatric Unit.” International Journal of Gerontology 8 (2): 56–59. doi:10.1016/j.ijge.2014.01.002.

[R4] Calder LA, Forster A, Nelson M, Leclair J, Perry J, Vaillancourt C, Hebert G, Cwinn A, Wells G, and Stiell I. 2010. “Adverse Events among Patients Registered in High-Acuity Areas of the Emergency Department: A Prospective Cohort Study.” CJEM 12 (5): 421–430. doi:10.1017/s1481803500012574. 20880432

[R5] Calder LA, Forster AJ, Stiell IG, Carr LK, Perry JJ, Vaillancourt C, and Brehaut J. 2012. “Mapping out the Emergency Department Disposition Decision for High-Acuity Patients.” Annals of Emergency Medicine 60 (5): 567–576.e4. doi:10.1016/j.annemergmed.2012.04.013. 22699018

[R6] Capan M, Pigeon J, Marco D, Powell J, and Groner K. 2018. “We All Make Choices: A Decision Analysis Framework for Disposition Decision in the ED.” The American Journal of Emergency Medicine 36 (3): 450–454. doi:10.1016/j.ajem.2017.11.018. 29174450

[R7] Carayon P 2009. “The Balance Theory and the Work System Model … Twenty Years Later.” International Journal of Human-Computer Interaction 25 (5): 313–327. doi:10.1080/10447310902864928.

[R8] CarayonP, Schoofs HundtA, KarshBT, GursesAP, AlvaradoCJ, SmithM, and Flatley BrennanP. 2006. “Work System Design for Patient Safety: The SEIPS Model.” Quality & Safety in Health Care 15 (suppl 1): i50–i58. doi:10.1136/qshc.2005.015842.17142610 PMC2464868

[R9] CarayonP, WetterneckT, Rivera-RodriguezAJ, HundtA, HoonakkerP, HoldenRJ, and GursesAP. 2014. “Human Factors Systems Approach to Healthcare Quality and Patient Safety.” Applied Ergonomics 45 (1): 14–25. doi:10.1016/j.apergo.2013.04.023.23845724 PMC3795965

[R10] Carman E-M, Fray M, and Waterson P. 2021. “Facilitators and Barriers of Care transitions - Comparing the Perspectives of Hospital and Community Healthcare Staff.” Applied Ergonomics 93: 103339. doi:10.1016/j.apergo.2020.103339. 33611077

[R11] Chimi C 2022. “The Likert Scale: A Proposal for Improvement Using QuasiContinuous Variables.”

[R12] CunninghamCeara Tess, QuanHude, HemmelgarnBrenda, NoseworthyTom, BeckCynthia A., DixonElijah, SamuelSusan, GhaliWilliam A., SykesLindsay L., and NathalieJetté. 2015. “Exploring Physician Specialist Response Rates to Web-Based Surveys.” BMC Medical Research Methodology 15 (1): 32. doi:10.1186/s12874-015-0016-z.25888346 PMC4404667

[R13] DanielsLM, SoritaA, KashiwagiDT, OkuboM, SmallE, PolleyEC, and SawatskyAP. 2018. “Characterizing Potentially Preventable Admissions: A Mixed Methods Study of Rates, Associated Factors, Outcomes, and Physician Decision-Making.” Journal of General Internal Medicine 33 (5): 737–744. Electronic). doi:10.1007/s11606-017-4285-6.29340940 PMC5910342

[R14] Diamond IR, Grant RC, Feldman BM, Pencharz PB, Ling SC, Moore AM, and Wales PW. 2014. “Defining Consensus: A Systematic Review Recommends Methodologic Criteria for Reporting of Delphi Studies.” Journal of Clinical Epidemiology 67 (4): 401–409. doi:10.1016/j.jclinepi.2013.12.002. 24581294

[R15] DyrstadDN, TestadI, and StormM. 2015. “Older Patients’ Participation in Hospital Admissions through the Emergency Department: An Interview Study of Healthcare Professionals.” BMC Health Services Research 15 (1): 475. doi:10.1186/s12913-015-1136-1.26486306 PMC4617984

[R16] Emerson P, Brooks D Fau-Quasim T, Quasim T, Fau-Puxty A, Puxty A, Fau-Kinsella J, Kinsella J, Fau Lowe DJ, and Lowe DJ. 2017. “Factors Influencing Intensive Care Admission: A Mixed Methods Study of EM and ICU.” European Journal of Emergency Medicine 24 (1): 29–35. doi:10.1097/MEJ.0000000000000300. 27984369

[R17] FernandoSM, RochwergB, ReardonPM, ThavornK, SeelyAJE, PerryJJ, BarnabyDP, TanuseputroP, and KyeremantengK. 2018. “Emergency Department Disposition Decisions and Associated Mortality and Costs in ICU Patients with Suspected Infection.” Critical Care (London, England) 22 (1): 172. doi:10.1186/s13054-018-2096-8.29976238 PMC6034286

[R18] Gorski JK, Batt RJ, Otles E, Shah MN, Hamedani AG, and Patterson BW. 2017. “The Impact of Emergency Department Census on the Decision to Admit.” Academic Emergency Medicine 24 (1): 13–21. doi:10.1111/acem.13103. 27641060

[R19] Graham C 2010. “Hearing the Voices of General Staff: A Delphi Study of the Contributions of General Staff to Student Outcomes.” Journal of Higher Education Policy and Management 32 (3): 213–223. doi:10.1080/13600801003743315.

[R20] Habibi A, Sarafrazi A, and Izadyar S. 2014. “Delphi Technique Theoretical Framework in Qualitative.” International Journal of Engineering Science 3: 8–13.

[R21] Hasson F, Keeney S, and McKenna H. 2000. “Research Guidelines for the Delphi Survey Technique.” Journal of Advanced Nursing 32 (4): 1008–1015. doi:10.1046/j.1365-2648.2000.t01-1-01567.x. 11095242

[R22] Hay G, Klonek F, and Parker S. 2020. “Diagnosing Rare Diseases: A Sociotechnical Approach to the Design of Complex Work Systems.” Applied Ergonomics 86: 103095. doi:10.1016/j.apergo.2020.103095. 32342886

[R23] Helmer-Hirschberg O 1967. “Analysis of the Future: The Delphi Method.” RAND Corporation. https://www.rand.org/pubs/papers/P3558.html

[R24] Hendrick HW, and Kleiner BM. 2001. “Macroergonomics - An Introduction to Work System Design.”

[R25] HoldenRJ, CarayonP, Fau-GursesAP, Gurses Ap Fau-HoonakkerP, HoonakkerP, Fau-HundtAS, Hundt As Fau-OzokAA, OzokAJ Rivera-RodriguezAa Fau, and Rivera-RodriguezAJ. 2013. “SEIPS 2.0: A Human Factors Framework for Studying and Improving the Work of Healthcare Professionals and Patients.” Ergonomics 56 (11): 1669–1686. doi:10.1080/00140139.2013.838643.24088063 PMC3835697

[R26] Holden R, and Carayon P. 2021. “SEIPS 101 and Seven Simple SEIPS Tools.” BMJ Quality & Safety 30 (11): 901–910. doi:10.1136/bmjqs-2020-012538. PMC8543199 34039748

[R27] HoldenR, SchubertC, and MickelsonR. 2015. “The Patient Work System: An Analysis of Self-Care Performance Barriers among Elderly Heart Failure Patients and Their Informal Caregivers.” Applied Ergonomics 47: 133–150. doi:10.1016/j.apergo.2014.09.009.25479983 PMC4258227

[R28] Hsu C-C, and Sandford B. 2007. “The Delphi Technique: Making Sense Of Consensus.” Practical Assessment, Research and Evaluation 12.

[R29] Hutchins E 1995. Cognition in the Wild. Cambridge, MA: MIT Press.

[R30] Hutchins E 2000. “Distributed Cognition.” International Encyclopedia of the Social and Behavioral Sciences 138.

[R31] Keeney S, Hasson F, and McKenna H. 2006. “Consulting the Oracle: Ten Lessons from Using the Delphi Technique in Nursing Research.” Journal of Advanced Nursing 53 (2): 205–212. doi:10.1111/j.1365-2648.2006.03716.x. 16422719

[R32] Klein G 2008. “Naturalistic Decision Making.” Human Factors 50 (3): 456–460. doi:10.1518/001872008X288385. 18689053

[R33] Leplat J 1989. “Error Analysis, Instrument and Object of Task Analysis.” Ergonomics 32 (7): 813–822. doi:10.1080/00140138908966844.

[R34] Lin MP, Natsui S, Sinnette C, Ball A, Weissman JS, and Schuur JD. 2018. “Facilitators and Barriers to Reducing Emergency Department Admissions for Chest Pain: A Qualitative Study.” Critical Pathways in Cardiology 17 (4): 201–207. doi:10.1097/HPC.0000000000000145. 30418250

[R35] Lippa KD, Feufel MA, Robinson FE, and Shalin VL. 2017. “Navigating the Decision Space: Shared Medical Decision Making as Distributed Cognition.” Qualitative Health Research 27 (7): 1035–1048. doi:10.1177/1049732316665347. 27557927

[R36] Loughlin KG, and Moore LF. 1979. “Using Delphi to Achieve Congruent Objectives and Activities in a Pediatrics Department.” Academic Medicine 54 (2): 101–106. https://journals.lww.com/academicmedicine/Fulltext/1979/02000/Using_Delphi_to_achieve_congruent_objectives_and.6.aspx. doi:10.1097/00001888-197902000-00006. 762686

[R37] McKenna H 1994. “The Delphi Technique: A Worthwhile Research Approach for Nursing?” Journal of Advanced Nursing 19 (6): 1221–1225. doi:10.1111/j.1365-2648.1994.tb01207.x. 7930104

[R38] McKenna H, and Hasson F. 2002. “A Study of Skill Mix Issues in Midwifery: A Multimethod Approach.” Journal of Advanced Nursing 37 (1): 52–61. doi:10.1046/j.1365-2648.2002.02058.x. 11784398

[R39] Nelson D, Washton D, Fau Jeanmonod R, and Jeanmonod R. 2013. “Communication Gaps in Nursing Home Transfers to the ED: impact on Turnaround Time, Disposition, and Diagnostic Testing.” The American Journal of Emergency Medicine 31 (4): 712–716. doi:10.1016/j.ajem.2012.11.024. 23380123

[R40] Nugus P, Holdgate A, Fry M, Forero R, McCarthy S, and Braithwaite J. 2011. “Work Pressure and Patient Flow Management in the Emergency Department: Findings From an Ethnographic Study.” Academic Emergency Medicine 18 (10): 1045–1052. doi:10.1111/j.1553-2712.2011.01171.x. 21996069

[R41] Pope I, Burn H, Ismail SA, Harris T, and McCoy D. 2017. “A Qualitative Study Exploring the Factors Influencing Admission to Hospital from the Emergency Department.” BMJ Open 7 (8): e011543. doi:10.1136/bmjopen-2016-011543. PMC5577896 28851767

[R42] Powell C 2003. “Methodological Issues In Nursing Research.” The Delphi technique: myths and realities. 41. 10.1046/j.1365-2648.2003.02537.x 12581103

[R43] Preston CC, and Colman AM. 2000. “Optimal Number of Response Categories in Rating Scales: reliability, Validity, Discriminating Power, and Respondent Preferences.” Acta Psychologica 104 (1): 1–15. doi:10.1016/s0001-6918(99)00050-5. 10769936

[R44] ProbstMA, KanzariaHK, SchoenfeldEM, MenchineMD, BreslinM, WalshC, MelnickER, and HessEP. 2017. “Shared Decisionmaking in the Emergency Department: A Guiding Framework for Clinicians.” Annals of Emergency Medicine 70 (5): 688–695. doi:10.1016/j.annemergmed.2017.03.063.28559034 PMC5834305

[R45] ProbstMA, KanzariaHK, HoffmanJR, MowerWR, MoheimaniRS, SunBC, and QuigleyDD. 2015. “Emergency Physicians’ Perceptions and Decision-Making Processes Regarding Patients Presenting with Palpitations.” The Journal of Emergency Medicine 49 (2): 236–243.e2. doi:10.1016/j.jemermed.2015.02.013.25943288 PMC4522216

[R46] RanceSA-O, WestlakeDA-O, BrantH, HolmeI, EndacottR, PinkneyJ, and ByngR. 2020. “Admission Decision-Making in Hospital Emergency Departments: The Role of the Accompanying Person.” Global Qualitative Nursing Research 7: 2333393620930024. doi:10.1177/2333393620930024.32596418 PMC7303774

[R47] RingerT, DoughertyM, McQuownC, MeladyD, OuchiK, SoutherlandL, and HoganT. 2018. “White Paper-Geriatric Emergency Medicine Education: Current State, Challenges, and Recommendations to Enhance the Emergency Care of Older Adults.” AEM Education and Training 2 (Suppl 1): S5–S16. doi:10.1002/aet2.10205.30607374 PMC6304282

[R48] Rowe G, and Wright G. 1999. “The Delphi Technique as a Forecasting Tool: Issues and Analysis.” International Journal of Forecasting 15 (4): 353–375. doi:10.1016/S0169-2070(99)00018-7.

[R49] Rutkowski RA, Pulia MS, Jaeger L, Loveless E, Patterson BW, Shah MN, Hoonakker P, Carayon P, Smith M, and Werner NE. 2025. “A Work Systems Approach to Characterizing Disposition Decision-Making under Low and High Demand.” Applied Ergonomics. 10.1016/j.apergo.2026.104737 PMC13394379 41564600

[R50] Rutkowski RA, Pulia MS, Salwei M, Loveless E, Jaeger L, Rawson M, Wust K, Hoonakker P, King B, Shah MN, Patterson BW, Dáil PW, Smith M, Carayon P, & Werner NE. 2022. Understanding Disposition Decision-Making for Older Adults as It Occurs within the Emergency Department Work System. In Proceedings of the Human Factors and Ergonomics Society Annual Meeting, Vol. 66, No. 1, 1156–1157. Sage CA: Los Angeles, CA: SAGE Publications. doi:10.1177/1071181322661509.

[R51] Rutkowski RA, Salwei M, Barton H, Wust K, Hoonakker P, Brenny-Fitzpatrick M, King B, Shah MN, Pulia MS, Patterson BW, Dáil P. v., Smith M, Carayon P, & Werner NE. 2020, December Physician Perceptions of Disposition Decision-Making for Older Adults in the Emergency Department: A Preliminary Analysis. In Proceedings of the Human Factors and Ergonomics Society Annual Meeting, Vol. 64, No. 1, 648–652. Sage CA: Los Angeles, CA: SAGE Publications. doi:10.1177/1071181320641148. PMC8256446 34234398

[R52] Rutkowski Rachel A., Scheer Eleanore, Carlson Claire, Parks Reid, Pulia Michael S., Patterson Brian W., Shah Manish N., Hoonakker Peter L. T., Carayon Pascale, Smith Maureen, Christensen Leslie A., and Werner Nicole E.. 2023. “A Scoping Review of Work System Elements That Influence Emergency Department Disposition Decision-Making.” Human Factors in Healthcare 4: 100059. doi:10.1016/j.hfh.2023.100059.

[R53] Salwei ME, Barton H, Werner NE, Rutkowski R, Hoonakker PL, Wust K, Shah MN, Patterson BW, Pulia MS, Hamedani AG, Smith M, King B, Dail PW, & Carayon P. 2020, December Identifying Roles in Older Adults’ Emergency Department Transitions. In Proceedings of the Human Factors and Ergonomics Society Annual Meeting, Vol. 64, No. 1, 685–689. Sage CA: Los Angeles, CA: SAGE Publications. doi:10.1177/1071181320641159. PMC8256446 34234398

[R54] Samaras N, Chevalley T, Samaras D, and Gold G. 2010. “Older Patients in the Emergency Department: A Review.” Annals of Emergency Medicine 56 (3): 261–269. doi:10.1016/j.annemergmed.2010.04.015. 20619500

[R55] SchechtmanM, KocherKE, NypaverMM, HamJJ, ZochowskiMK, and MacyML. 2019. “Michigan Emergency Department Leader Attitudes Toward and Experiences With Clinical Pathways to Guide Admission Decisions: A Mixed-Methods Study.” Academic Emergency Medicine: official Journal of the Society for Academic Emergency Medicine 26 (4): 384–393. doi:10.1111/acem.13555.30112831 PMC12702504

[R56] Shanafelt Tait D., Bradley Katharine A., Wipf Joyce E., and Back Anthony L.. 2002. “Burnout and Self-Reported Patient Care in an Internal Medicine Residency Program.” Annals of Internal Medicine 136 (5): 358–367. doi:10.7326/0003-4819-136-5-200203050-00008. 11874308

[R57] SiddiqueSM, Lane-FallM, McConnellMJ, JakheteN, CrismaleJ, PorgesS, KhungarV, MehtaSJ, GoldbergD, LiZ, SchianoT, ReganL, OrloskiC, and SheaJA. 2018. “Exploring Opportunities to Prevent Cirrhosis Admissions in the Emergency Department: A Multicenter Multidisciplinary Survey.” Hepatology Communications 2 (3): 237–244. doi:10.1002/hep4.1141.29507899 PMC5831018

[R58] Skulmoski G, Hartman F, and Krahn J. 2007. “The Delphi Method for Graduate Research.” JITE 6: 1–21. doi:10.28945/199.

[R59] Stiell Andrew, Forster Alan J., Stiell Ian G., and Carl van Walraven. 2003. “Prevalence of Information Gaps in the Emergency Department and the Effect on Patient Outcomes.” CMAJ: Canadian Medical Association Journal = Journal de L’Association Medicale Canadienne 169 (10): 1023–1028. PMC236227 14609971

[R60] StuckA, CrowleyC, MartinezT, WittgroveA, BrennanJJ, ChanTC, and CastilloEM. 2017. “Perspectives on Home-Based Healthcare as an Alternative to Hospital Admission After Emergency Treatment.” The Western Journal of Emergency Medicine 18 (4): 761–769. doi:10.5811/westjem.2017.3.32348.28611899 PMC5468084

[R61] Turner AJ, Anselmi L, Lau Y-S, and Sutton M. 2020. “The Effects of Unexpected Changes in Demand on the Performance of Emergency Departments.” Health Economics 29 (12): 1744–1763. doi:10.1002/hec.4167. 32978879

[R62] Vagias Wade M. 2006. Likert-Type Scale Response Anchors. Clemson International Institute for Tourism & Research Development, Department of Parks, Recreation and Tourism Management. Clemson, SC: Clemson University.

[R63] VanGeest JB, Johnson TP, and Welch VL. 2007. “Methodologies for Improving Response Rates in Surveys of Physicians: A Systematic Review.” Evaluation & the Health Professions 30 (4): 303–321. doi:10.1177/0163278707307899. 17986667

[R64] Wears RL, Woloshynowych M, Fau-Brown R, Brown CA Vincent R Fau, and Vincent CA. 2010. “Reflective Analysis of Safety Research in the Hospital Accident & Emergency Departments.” Applied Ergonomics 41 (5): 695–700. 0. doi:10.1016/j.apergo.2009.12.006. 20089245

[R65] WernerN, PonnalaS, DoutchevaN, and HoldenR. 2021. “Human Factors/Ergonomics Work System Analysis of Patient Work: State of the Science and Future Directions.” International Journal for Quality in Health Care 33 (Supplement_1): 60–71. doi:10.1093/intqhc/mzaa099.33432984 PMC7802067

[R66] Westert Gert P., and Groenewegen Peter P.. 1999. “Medical Practice Variations: changing the Theoretical Approach.” Scandinavian Journal of Public Health 27 (3): 173–180. doi:10.1177/14034948990270030801. 10482075

[R67] Wright B, Martin GP, Ahmed A, Banerjee J, Mason S, and Roland D. 2018. “How the Availability of Observation Status Affects Emergency Physician Decisionmaking.” Annals of Emergency Medicine 72 (4): 401–409. doi:10.1016/j.annemergmed.2018.04.023. 29880439

